# Trained Nurses' Fund

**Published:** 1887-06-18

**Authors:** 


### TRAINED NURSES' FUND.

We continue to receive many requests for particulars—definite details regarding this Fund. We are glad that matrons, sisters, and other ladies are so business-like in their views. But now we desire that they will be business like in their practice as well. We have frequently stated that no definite steps can be taken until at least two thousand names are sent in. Up to the present time we have received rather less than three hundred. Our scheme, prepared by an eminent actuary, is quite ready; but, like a ship ready to be launched, it must wait for the tide—the high-water mark of two thousand names. Nurses and others need not be afraid of sending in their names. They do not incur any responsibility whatever by so doing; but simply signify their willingness to become members of the Fund hereafter, provided its conditions meet their approval. If the matrons, sisters, and nurses do their part there is no reason why the scheme, with all its details, should not be published within a few weeks of the present time, and established and in full working order before Christmas.—Ed. T. H.

